# Developmental competence of alpaca oocytes matured *in vivo* with seminal plasma and following additional *in vitro* maturation

**DOI:** 10.1590/1984-3143-AR2024-0138

**Published:** 2025-12-19

**Authors:** Nancy Milagros Silva Huanca, Víctor Hugo Cornelio Díaz, Luis Antonio Auqui Rojas, Alexis Ivan Huaman Apaza, Wilfredo Huanca López

**Affiliations:** 1 Laboratorio de Reproducción Animal, Facultad de Medicina Veterinaria, Universidad Nacional Mayor de San Marcos, Lima, Perú

**Keywords:** alpaca, seminal plasma, in vivo, oocyte maturation, developmental competence

## Abstract

The aim of this study was to evaluate the effects of *in vivo* oocyte maturation using seminal plasma alone or in combination with an additional period of *in vitro* maturation (IVM) on the developmental competence of alpaca oocytes. The experiment was conducted in Lima, Peru, with twelve adult female alpacas. Follicular ablation of the dominant follicle was performed to initiate a new follicular wave. After 36 hours, a superstimulation protocol with 750 IU of eCG was administered intramuscularly (IM). Four days later, 2 mL of seminal plasma was administered IM to promote *in vivo* oocyte maturation. Cumulus-oocyte complexes (COCs) were retrieved via ovum pick-up 20 hours post-treatment, morphologically evaluated, and allocated into three groups: (T1) no additional IVM, (T2) 12 hours of additional IVM, and (T3) 18 hours of additional IVM. Oocyte developmental competence was assessed using a 26 µM brilliant cresyl blue (BCB) staining protocol for 90 minutes. COCs were classified as BCB positive (blue ooplasm) or BCB negative (unstained ooplasm) and subsequently denuded and fixed for nuclear maturation assessment via orcein staining. No significant differences (p=0.14) were observed in the percentage of expanded COCs across groups (71.4%, 81.8%, and 54.6% for T1, T2, and T3, respectively). The proportion of COCs reaching the metaphase II (MII) stage was higher (p<0.05) in T3 (54.6%), while the developmental competence rate was greatest (p<0.05) in T2 (100%). However, no differences (p=0.21) were detected in the proportion of BCB positive MII stage COCs across groups. In conclusion, alpaca oocytes matured *in vivo* with seminal plasma require 12 to 18 hours of IVM to achieve optimal nuclear maturation and developmental competence.

## Introduction

South American camelids are species of significant socio-economic importance for Andean populations due to their resilience and adaptation to altitudes exceeding 3800 meters above sea level. Despite these advantages, their productivity is often constrained by poor genetic quality, which has driven interest in advancing reproductive biotechnologies to enhance genetic improvement. Among these, *in vitro* embryo production through *in vitro* fertilization (IVF) has emerged as a promising tool, although its application in South American camelids has been met with challenges (Huanca, 2015). Currently, blastocyst rates achieved through *in vitro* embryo production in these species range from 20% to 35% (Trasorras et al., 2014), with oocyte quality and developmental competence being key factors limiting success (Moawad et al., 2020).

Traditional methods for cumulus-oocyte complexes (COCs) selection rely on morphological assessment, which, while straightforward, can be subjective and inaccurate. Contemporary techniques using vital stains offer more precise and practical evaluations of oocyte quality, mainly in terms of maturation and developmental competence of oocytes (Ashry et al., 2015). Oocyte developmental competence—the capacity to resume meiosis, undergo fertilization, and develop into a blastocyst—can be measured by enzymatic activity (Sirard et al., 2006). Brilliant cresyl blue (BCB) staining is one such method, measuring glucose-6-phosphate dehydrogenase (G6PDH) activity. As oocytes mature and their G6PDH activity declines, BCB positive oocytes stain blue, indicating their competence (Opiela and Kitska-Ksiizkiewicz, 2013).

*In vivo* maturation techniques involving gonadotropin-releasing hormone (GnRH) or luteinizing hormone (LH) analogs mimic the natural LH surge preceding ovulation, which facilitates nuclear maturation and progression to the metaphase II (MII) stage. The LH surge also induces expansion of cumulus cells, a morphological change associated with oocyte maturation (Roelen, 2019). In ruminants, Rizos et al. (2002) reported *in vivo* oocyte maturation with LH for 20 hours prior to COCs collection from animals with an ovarian superstimulation protocol yield superior embryonic development rates at day 7 post IVF compared to 24 hours *in vitro* maturation (IVM) of oocytes from 2-6 mm and ≥6 mm follicles (49% vs 32% and 39%, respectively). Another study in ovarian superstimulated cattle (Matoba et al., 2014) established that it is optimal to perform ovum pick-up (OPU) maximum up to 26 hours after *in vivo* maturation with GnRH to avoid losing the COCs due to the occurrence of ovulation, but 4 extra hours of IVM are necessary since it is only after 30 hours post *in vivo* maturation that LH levels increase, which would be causing nuclear oocyte maturation. In that same study, performing IVF of *in vivo* matured oocytes, a higher percentage of good quality blastocysts was obtained on day 9 (55%) compared to oocytes obtained from non-superstimulated cows matured *in vitro* for 24 hours (36%).

Reports on *in vivo* oocyte maturation in South American camelids are scarce. In llamas, ovarian superstimulation followed by *in vivo* maturation with GnRH for 20 hours prior to surgical follicular aspiration yielded a 35% blastocyst rate at day 6 post IVF (Trasorras et al., 2014). Similarly, another study in llamas utilizing LH for *in vivo* oocyte maturation and ovum pick-up (OPU) reported 79% of oocytes reaching the MII stage determined by orcein staining (Ratto et al., 2005). In contrast, superstimulated dromedary camels exhibited higher MII rates after *in vivo* oocyte maturation with GnRH, achieving 82% and 91% for 26-27 and 28-29 hours of maturation, respectively (Wani and Skidmore, 2010).

In dromedaries, BCB staining has been used to assess oocyte quality, showing that oocytes from slaughterhouse ovaries classified as BCB positive had a higher proportion of MII stage oocytes (75%) compared to BCB negative oocytes (41%) after 36 hours of IVM (Fathi et al., 2017). In alpacas, a recent study (Vargas-Donayre et al., 2024) also found a significant difference in MII stage oocytes between BCB positive (29%) and BCB negative (12%) groups after 48 hours of IVM. However, these results remain lower than those reported in dromedaries, suggesting species-specific differences in oocyte competence. Despite these findings, alpaca-specific data on *in vivo* oocyte maturation and its impact on developmental competence are sparse, highlighting the need for further research in this area.

The β-nerve growth factor present in the seminal plasma of South American camelids plays a crucial role in inducing ovulation by stimulating a pre-ovulatory LH surge (Adams et al., 2005; Kershaw-Young et al., 2012). While the ovulation-inducing effects of seminal plasma are well-documented, its role in pre-ovulatory oocyte maturation and potential applications in assisted reproduction technologies are less understood.

Given this knowledge gap, the present study aims to evaluate whether seminal plasma can support *in vivo* oocyte maturation in alpacas and to determine if additional IVM is required to enhance developmental competence. Through a combination of morphological assessment, BCB staining, and nuclear maturation analysis, this approach offers a non-hormonal alternative with potential applications in assisted reproductive technologies for camelids.

## Methods

### Study design

This study was conducted during September and October 2024 at the Animal Reproduction Laboratory of the Faculty of Veterinary Medicine, Universidad Nacional Mayor de San Marcos, Lima, Peru. Animals were maintained under uniform conditions and fed alfalfa hay. Animal procedures were reviewed and approved by the Ethics and Animal Welfare Committee of the Faculty of Veterinary Medicine of the National University of San Marcos (approval number CEBA 2021-23). Plastic materials were sourced from Falcon (USA), and chemical reagents were obtained from Sigma (USA), unless otherwise specified.

### Seminal plasma collection

Seminal plasma was obtained from four adult male alpacas (4–7 years old) through a protocol adapted from Adams et al. (2005). Ejaculates were collected using an artificial vagina adapted for sheep, with a frequency of twice a week over one month preceding the experimental phase. Semen was diluted 1:1 (v/v) with phosphate-buffered saline and subjected to mechanical passage through a 21-gauge needle to reduce viscosity. The sample was centrifuged at 1500 g for 20 minutes, and the process was repeated to ensure the removal of sperm cells. The resulting supernatants were pooled, supplemented with 1 µg/mL gentamicin sulfate, and stored at -20°C until use.

### Animal management and i*n vivo* oocyte maturation

Twelve adult non-pregnant female alpacas (4–6 years old), without reproductive abnormalities, and with a history of at least one previous birth, were selected. Pre-ovulatory follicles ≥7 mm in diameter were confirmed by transrectal ultrasonography using an EXAPAD Mini Ultrasound (IMV Imaging, USA) with a 7.5 MHz linear transducer. To mitigate the inhibitory effects of the dominant follicle, follicular ablation of these pre-ovulatory structures was performed using an 18-gauge needle guided by a 7.5 MHz convex transducer. Subsequently, a superstimulation protocol was initiated 36 hours later with the intramuscular (IM) administration of 750 IU of equine chorionic gonadotropin (eCG) (Syntex, Argentina) (Huanca et al., 2020). Four days post-eCG administration, 2 mL of seminal plasma was administered IM to induce *in vivo* oocyte maturation. The ovarian response was assessed by transrectal ultrasonography using a 7.5 MHz linear transducer immediately before oocyte collection.

### Obtaining and morphological classification of cumulus-oocyte complexes

After 20 hours of seminal plasma administration, COCs were retrieved using OPU as described by Brogliatti et al. (2000). Low-grade epidural anesthesia was administered beforehand by injecting 1.5 ml of 2% lidocaine without epinephrine into the sacro-coccygeal space. A 7.5 MHz convex transducer and an 18-gauge needle, both previously disinfected with a potassium peroxymonosulfate and sodium chloride solution, were used for the procedure. The transducer was introduced transvaginally to the anterior fornix, with the perianal area of the female thoroughly cleaned prior to insertion. To stabilize the ovary containing the target follicles, the free hand was placed into the rectum. Under ultrasound guidance, the dominant follicle was positioned centrally on the EXAPAD mini ultrasound screen, and the aspiration needle was advanced through its guide on the transducer until it penetrated the follicle wall. A vacuum pump (Minitube, Germany) generated constant pressure at -55 mm Hg to aspirate the follicular contents, which were transferred directly into test tubes containing 5 mL of OPU recovery medium (Minitube, Germany) maintained at 37°C. The aspirated contents were passed through an oocyte strainer filter (WTA, Brazil) and the filter was washed in tempered 90x15 mm Petri dishes at 37°C. COCs were observed under a 20x stereomicroscope and classified into three categories based on their morphology according to Ratto et al. (2005): with expanded (loose cumulus), compact (≥ 2 layers of cumulus cells), or partially denuded (with minimal cumulus surrounding). COCs were randomly allocated into three groups: (T1) no additional IVM, (T2) 12 hours of additional IVM, and (T3) 18 hours of additional IVM.

### *In vitro* maturation of oocytes

For groups requiring IVM, COCs were rinsed twice in drops of commercial IVM medium (Stroebech, Denmark) and transferred into culture vials containing 800 µL of pre-equilibrated IVM medium. The vials were incubated at 38.5°C with 6% CO_2_ for the designated duration per group protocol.

### Brilliant cresyl blue staining

Oocyte competence was evaluated via BCB staining, following the protocol established by Ashry et al. (2015). COCs were incubated in 500 µL of 26 µM BCB stain diluted in DPBS (Dulbecco’s phosphate-buffered saline) supplemented with 0.4% bovine serum albumin (BSA) for 90 minutes at 38.5°C in a humidified atmosphere with 5% CO_2_. Following incubation, COCs were rinsed in supplemented DPBS and classified as: BCB positive (competent; with blue ooplasm) or BCB negative (non-competent; with unstained ooplasm).

### Nuclear maturation assessment

COCs were grouped according to their BCB classification. Each group underwent denudation by vortexing at 2200 rpm for 2 minutes in a polystyrene tube containing DPBS supplemented with BSA and hyaluronidase. The denuded oocytes were fixed in a solution of ethanol and acetic acid (3:1, v/v) for 36 hours. Fixed oocytes were stained with 1% orcein and examined under a phase-contrast microscope to determine their nuclear maturation stage.

### Statistical analysis

The morphological quality of COCs, nuclear maturation rates, and developmental competence rates were analyzed using the Chi-square test. This analysis assessed whether the observed distributions of outcomes aligned with expected distributions, determining the significance of differences among treatment groups.

## Results

**Figure 1:**
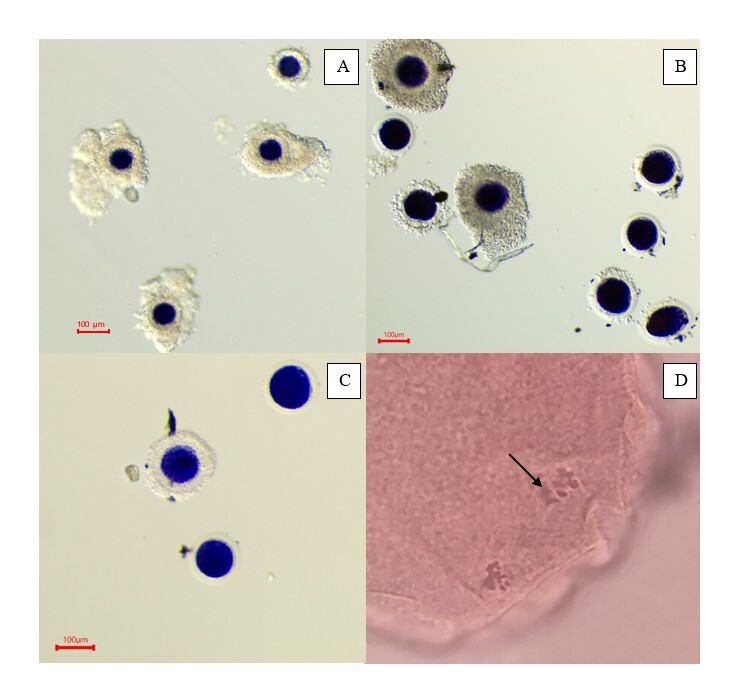
Alpaca oocytes exhibiting BCB positivity and metaphase II stage after various maturation protocols: BCB positivity only 20 hours of *in vivo* maturation (A), BCB positivity 12 hours of additional IVM (B), BCB positivity 18 hours of additional IVM (C) and metaphase II stage (D) with a metaphase plate (arrow).

A total of 72 COCs were collected from 94 follicles aspirated through OPU with a COC recovery rate of 76% and 7.8 COCs collected per alpaca. No significant differences (p=0.14) were observed in the percentage of expanded COCs across groups (Table 1). In T2, all expanded and compact COCs were BCB positive, representing the highest percentage of BCB positive expanded COCs among the groups. In contrast, the lowest percentage of BCB positive compact COCs was observed in T3 (14.3%) (p < 0.05; Table 1). Regarding nuclear maturation, the percentage of COCs reaching MII stage was significantly higher in T3 (54.6%) (p < 0.05; Table 2). However, no differences (p=0.21) were detected in the proportion of BCB positive MII stage COCs across groups (Figure 1, Table 2). Developmental competence assessed by BCB staining showed the highest rate of BCB-positive COCs in T2 (100%), which was significantly different from T1 (39.3%) and T3 (13.6%) (p < 0.05; Table 2).

**Table 1:** Morphological classification and distribution of COCs BCB+.

**Groups**	**COCs collected**		**Expanded COCs**		**Partially denuded COCs**		**Compact COCs**	
	**BCB+ (%)**	**Total**	**BCB+ (%)**	**Total (%)**	**BCB+ (%)**	**Total (%)**	**BCB+ (%)**	**Total (%)**
T1	11 (39.3^a^)	28	6/20 (30.0^a^)	20 (71.4)	0/3 (0)	3 (10.7)	5/5 (100.0^a^)	5 (17.9)
T2	22 (100.0^b^)	22	18/18 (100.0^b^)	18 (81.8)	0	0	4/4 (100.0^a^)	4 (18.2)
T3	3 (13.6 ^a^)	22	2/12 (16.7^a^)	12 (54.6)	0/3 (0)	3 (13.6)	1/7 (14.3^b^)	7 (31.8)

T1: only 20 h *in vivo* maturation; T2: 12 hours of additional IVM; T3: 18 hours of additional IVM. The same columns with different superscripts differ significantly (p<0.05).

**Table 2:** Nuclear maturation stage of COCs and distribution of COCs BCB+ reaching MII stage.

**Groups**	**COCs collected**	**GV stage**	**MI stage**	**MII stage**	**Degenerated COCs**	**COCs BCB+**	**COCs BCB+ MII stage**
	**n**	**n (%)**	**n (%)**	**n (%)**	**n (%)**	**n (%)**	**n (%)**
T1	28	6 (21.4)	7 (25.0)	2 (7.1^a^)	13 (46.4)	11/28 (39.3^a^)	2/11 (18.2)
T2	22	5 (22.7)	6 (27.3)	4 (18.2^a^)	7 (31.8)	22/22 (100.0^b^)	4/22 (18.2)
T3	22	0	5 (22.7)	12 (54.6^b^)	5 (22.7)	3/22 (13.6^a^)	2/3 (66.7)

T1: only 20 h *in vivo* maturation; T2: 12 hours of additional IVM; T3: 18 hours of additional IVM. The same columns with different superscripts differ significantly (p<0.05).

## Discussion

In superstimulated South American camelids, COCs recovery rates using OPU procedures have been reported to range between 70% and 80% (Berland et al., 2011; Huanca et al., 2020; Ratto et al., 2005). Our study achieved a similar recovery rate of 76%. Regarding ovarian superstimulation protocols, previous studies have found no significant differences in the number of COCs obtained when comparing the administration of 200 mg of follicle-stimulating hormone to 1000 IU of eCG per llama (10.7 vs. 11.2, respectively) (Ratto et al., 2005). In contrast, our application of 750 IU of eCG per alpaca yielded a lower number of COCs per animal (7.8), indicating that eCG dosage optimization may be species-specific.

Oocyte maturation is frequently evaluated based on morphological criteria, such as the presence of COCs with expanded cumulus. In our study, no significant differences were observed among the groups in the proportion of COCs with expanded cumulus. These findings contrast with earlier studies in llamas, which reported higher proportions of COCs with expanded cumulus after 20 hours of *in vivo* maturation using LH (94%) (Ratto et al., 2005) or GnRH (100%) (Trasorras et al., 2014). However, in our study T2 exhibited the highest proportion of BCB-positive oocytes, all of which were classified as morphologically expanded or compact. These differences could reflect the inherent subjectivity of morphological criteria in assessing the quality of *in vivo*-matured alpaca oocytes and also suggests that the 12-hour IVM period may coincide with a favorable window for metabolic activation and competence acquisition.

This study investigates an alternative *in vivo* oocyte maturation method in alpacas, utilizing seminal plasma as a non-hormonal approach. Recognizing that alpaca oocytes typically require 28 to 36 hours for *in vitro* maturation, the present study incorporated an additional in vitro maturation (IVM) period subsequent to *in vivo* treatment. The proportion of COCs reaching the MII stage following *in vivo* maturation with seminal plasma alone (T1 group) was merely 7%, a figure significantly lower than the 64% reported by Ratto et al. (2005) with LH treatment. However, the results from the T3 group (55%) demonstrated a closer alignment with this previous finding, indicating that oocytes subjected to an additional 18 hours of IVM experienced enhanced meiotic progression. These data collectively suggest that while seminal plasma may initiate early maturation events, it is insufficient on its own to complete nuclear maturation, thereby underscoring the critical importance of an additional IVM period for achieving full nuclear competence.

The capacity to pre-screen for or enhance oocyte developmental competence is a critical factor for improving reproductive success (Roelen, 2019). In dromedary camels, a 75% maturation rate has been reported for BCB-positive COCs following 36 hours of IVM (Fathi et al., 2017). In the present study, the highest maturation rate for BCB-positive COCs was observed in Group T3 (67%). Our findings align with recent studies in alpacas, which also reported a 52% maturation rate after 36 hours of IVM (Vargas-Donayre et al., 2024).

In dromedary camels, collection of COCs by OPU after *in vivo* maturation has been extensively used for the production of calves by SCNT, showing promising developmental rates (Wani, 2021; Wani and Skidmore, 2010). While our study focuses on alpaca oocytes, these findings in dromedaries highlight the potential of *in vivo* maturation combined with OPU for advanced reproductive technologies in camelids. The comparison to these works suggests that alpaca oocytes, despite being camelids, may have species-specific requirements for optimal *in vivo* and *in vitro* maturation.

Our findings suggest that despite the systemic action of β-NGF in seminal plasma and its capacity to induce a more sustained LH secretion compared to GnRH, its potent local luteogenic effect may necessitate a longer duration for *in vivo* oocyte maturation than was feasible in the current experimental design, primarily due to the risk of ovum loss. Nevertheless, the precise mechanisms by which seminal plasma influences the complete oocyte maturation cascade, particularly the cytoplasmic changes crucial for developmental competence, remain to be fully elucidated. These mechanisms may indeed diverge from those driven purely by hormonal stimulations. This implies that while seminal plasma provides an initial physiological trigger, subsequent processes essential for achieving full competence, such as the reduction in G6PDH activity (indicated by BCB+ status) and progression to MII, might require an extended period or specific *in vitro* conditions.

Given the limited data on *in vivo* oocyte maturation in South American camelids, particularly alpacas, our findings offer significant insights into a novel, non-hormonal approach utilizing seminal plasma. Our data suggest that alpaca oocytes undergoing *in vivo* maturation with seminal plasma, even after experiencing a pre-ovulatory LH surge, require an additional 12 to 18 hours of IVM. This extended *in vitro* period appears crucial, bringing the total maturation time to 32 to 38 hours, to ensure complete developmental competence. This includes achieving both the final stages of nuclear maturation (MII) and optimal cytoplasmic maturation, as evidenced by the BCB+ status. Specifically, for BCB+ oocytes to reach the MII stage, this sufficient period allows for the necessary decrease in G6PDH activity, signaling full cytoplasmic competence, in parallel with nuclear progression. While seminal plasma can support *in vivo* oocyte maturation in alpacas, its effectiveness appears limited without this subsequent IVM. Collectively, these results strongly indicate that a combined approach, leveraging seminal plasma for *in vivo* priming followed by an additional 12-18 hour IVM period, is required to ensure the full developmental competence necessary for further reproductive processes in this species.

## Conclusion

In summary, alpaca oocytes matured *in vivo* with seminal plasma require 12 to 18 hours of IVM to attain optimal nuclear maturation and developmental competence. This combined approach may offer a viable alternative to hormonal induction protocols in assisted reproduction for South American camelids.

## Data Availability

Research data is only available upon request.
